# Supplementary material for: Artificial intelligence in orthopaedics: A scoping review
Source: PLoS One. 2021 Nov 23;16(11):e0260471. doi: 10.1371/journal.pone.0260471 (PMC8610245; doi:10.1371/journal.pone.0260471)
Supplement: S2 Table — (DOCX) [file pone.0260471.s002.docx]

| **S2 Table: Database search terms for Ovid - Embase and Medline** |
| --- |
| **1. Artificial intelligence.mp. or exp Artificial Intelligence/** |
| **2. Deep learning.mp. or exp Deep Learning/** |
| **3. Machine learning.mp. or exp Machine Learning/** |
| **4. exp "Neural Networks (Computer)"/ or Neural network*.mp.** |
| **5. Orthopedic Surgeons/ or Orthopedic Procedures/ or Orthop?edic*.mp.** |
| **6. 1 or 2 or 3 or 4** |
| **7. exp Arthroplasty/ or arthroplasty.mp.** |
| **8. Hip/ or Hip Joint/ or hip.mp.** |
| **9. Spine/ or spine.mp.** |
| **10. Shoulder/ or shoulder.mp.** |
| **11. exp Knee/ or knee.mp.** |
| **12. Spinal Stenosis/ or Spinal Cord Diseases/ or spinal.mp. or Spinal Cord Injuries/ or Spinal Fractures/ or Spinal Diseases/ or Spinal Nerve Roots/ or Spinal Fusion/ or Spinal Injuries/ or Spinal Cord Compression/ or Spinal Nerves/** |
| **13. wrist.mp. or exp Wrist/** |
| **14. elbow.mp. or exp Elbow/** |
| **15. Ankle/ or Ankle Joint/ or ankle.mp.** |
| **16. 5 or 7 or 8 or 9 or 10 or 11 or 12 or 13 or 14 or 15** |
| **17. 6 and 16** |
