# Supplementary material for: Artificial intelligence in orthopaedics: A scoping review
Source: PLoS One. 2021 Nov 23;16(11):e0260471. doi: 10.1371/journal.pone.0260471 (PMC8610245; doi:10.1371/journal.pone.0260471)
Supplement: S3 Table — (DOCX) [file pone.0260471.s003.docx]

| **S3 Table: Database search terms for Scopus** |
| --- |
| **( ( TITLE-ABS-KEY ( "machine learning" ) )  OR  ( TITLE-ABS-KEY ( "deep learning" ) )  OR  ( TITLE-ABS-KEY ( "artificial intelligence" ) )  OR  ( TITLE-ABS-KEY ( "neural network*" ) ) )  AND  ( ( TITLE-ABS-KEY ( orthop?edic* ) )  OR  ( TITLE-ABS-KEY ( arthroplasty ) )  OR  ( TITLE-ABS-KEY ( hip ) )  OR  ( TITLE-ABS-KEY ( knee ) )  OR  ( TITLE-ABS-KEY ( shoulder ) )  OR  ( TITLE-ABS-KEY ( ankle ) )  OR  ( TITLE-ABS-KEY ( spine ) )  OR  ( TITLE-ABS-KEY ( spinal ) ) )  AND  ( LIMIT-TO ( LANGUAGE ,  "English" ) )  AND  ( LIMIT-TO ( SRCTYPE ,  "j" )  OR  LIMIT-TO ( SRCTYPE ,  "p" ) )** |
