# Supplementary material for: Artificial intelligence in orthopaedics: A scoping review
Source: PLoS One. 2021 Nov 23;16(11):e0260471. doi: 10.1371/journal.pone.0260471 (PMC8610245; doi:10.1371/journal.pone.0260471)
Supplement: S4 Table — (DOCX) [file pone.0260471.s004.docx]

**S4 Table – Reference list of papers included in study**

[1] Abdelhameed EH, Sato N, Morita Y. Neural network-based shoulder instability diagnosis modelling for robot-assisted rehabilitation systems. Syst Sci Control Eng. 2015;3:514–523.

[2] Abedin J, Antony J, McGuinness K, et al. Predicting knee osteoarthritis severity: comparative modeling based on patient’s data and plain X-ray images. Sci Rep. 2019;9:5761.

[3] Adams M, Chen W, Holcdorf D, et al. Computer vs human: Deep learning versus perceptual training for the detection of neck of femur fractures. J Med Imaging Radiat Oncol. 2019;63:27–32.

[4] Adankon MM, Dansereau J, Labelle H, et al. Non invasive classification system of scoliosis curve types using least-squares support vector machines. Artif Intell Med. 2012;56:99–107.

[5] Akben SB. Importance of the shape and orientation of the spine and pelvis for the vertebral column pathologies diagnosis with using machine learning methods. Biomed Res. 2016;2016:S337–S342.

[6] Al-Helo S, Alomari RS, Ghosh S, et al. Compression fracture diagnosis in lumbar: A clinical CAD system. Int J Comput Assist Radiol Surg. 2013;8:461–469.

[7] Alomari RS, Corso JJ, Chaudhary V, et al. Toward a clinical lumbar CAD: Herniation diagnosis. Int J Comput Assist Radiol Surg. 2011;6:119–126.

[8] Ames CP, Smith JS, Pellisé F, et al. Artificial Intelligence Based Hierarchical Clustering of Patient Types and Intervention Categories in Adult Spinal Deformity Surgery: Towards a New Classification Scheme that Predicts Quality and Value. Spine (Phila Pa 1976). 2019;44:915–926.

[9] Ames CP, Smith JS, Pellisé F, et al. Development of predictive models for all individual questions of SRS-22R after adult spinal deformity surgery: a step toward individualized medicine. Eur Spine J. 2019;28:1998–2011.

[10] Anifah L, Purnama IKE, Hariadi M, et al. Osteoarthritis Classification Using Self Organizing Map Based on Gabor Kernel and Contrast-Limited Adaptive Histogram Equalization. Open Biomed Eng J. 2013;7:18–28.

[11] Aram P, Trela-Larsen L, Sayers A, et al. Estimating an Individual’s Probability of Revision Surgery after Knee Replacement: A Comparison of Modeling Approaches Using a National Data Set. Am J Epidemiol. 2018;187:2252–2262.

[12] Arslan YZ, Demirer RM, Palamar D, et al. Comparison of the data classification approaches to diagnose spinal cord injury. Comput Math Methods Med. 2012;2012:803980.

[13] Arvind V, Kim JS, Oermann EK, et al. Predicting surgical complications in adult patients undergoing anterior cervical discectomy and fusion using machine learning. Neurospine. 2018;15:329–337.

[14] Ashinsky BG, Coletta CE, Bouhrara M, et al. Machine learning classification of OARSI-scored human articular cartilage using magnetic resonance imaging. Osteoarthr Cartil. 2015;23:1704–1712.

[15] Ashinsky BG, Bouhrara M, Coletta CE, et al. Predicting early symptomatic osteoarthritis in the human knee using machine learning classification of magnetic resonance images from the osteoarthritis initiative. J Orthop Res. 2017;35:2243–2250.

[16] Athertya JS, Saravana Kumar G, Govindaraj J. Detection of Modic changes in MR images of spine using local binary patterns. Biocybern Biomed Eng. 2019;39:17–29.

[17] Atkinson EJ, Therneau TM, Melton LJ, et al. Assessing fracture risk using gradient boosting machine (GBM) models. J Bone Miner Res. 2012;27:1397–1404.

[18] Auloge P, Cazzato RL, Ramamurthy N, et al. Augmented reality and artificial intelligence-based navigation during percutaneous vertebroplasty: a pilot randomised clinical trial. Eur Spine J. 2020;29:1580–1589.

[19] Azimi P, Benzel EC, Shahzadi S, et al. Use of artificial neural networks to predict surgical satisfaction in patients with lumbar spinal canal stenosis: Clinical article. J Neurosurg Spine. 2014;20:300–305.

[20] Azimi P, Mohammadi HR, Benzel EC, et al. Use of artificial neural networks to predict recurrent lumbar disk herniation. J Spinal Disord Tech. 2015;28:E161–E165.

[21] Badgeley MA, Zech JR, Oakden-Rayner L, et al. Deep learning predicts hip fracture using confounding patient and healthcare variables. NPJ Digit Med. 2019;2:31.

[22] Badhiwala JH, Hachem LD, Merali Z, et al. Predicting outcomes after surgical decompression for mild degenerative cervical myelopathy: Moving beyond the mjoa to identify surgical candidates. Neurosurgery. 2020;86:565–573.

[23] Baka N, Leenstra S, Van Walsum T. Ultrasound Aided Vertebral Level Localization for Lumbar Surgery. IEEE Trans Med Imaging. 2017;36:2138–2147.

[24] Bayram F, Çakiroʇlu M. DIFFRACT: DIaphyseal Femur FRActure Classifier SysTem. Biocybern Biomed Eng. 2016;36:157–171.

[25] Bekta̧s F, Eken C, Soyuncu S, et al. Artificial neural network in predicting craniocervical junction injury: An alternative approach to trauma patients. Eur J Emerg Med. 2008;15:318–323.

[26] Belliveau T, Jette AM, Seetharama S, et al. Developing Artificial Neural Network Models to Predict Functioning One Year After Traumatic Spinal Cord Injury. Arch Phys Med Rehabil. 2016;97:1663-1668.e3.

[27] Bertsimas D, Masiakos PT, Mylonas KS, et al. Prediction of cervical spine injury in young pediatric patients: an optimal trees artificial intelligence approach. J Pediatr Surg. 2019;54:2353–2357.

[28] Beulah A, Sharmila TS, Pramod VK. Disc bulge diagnostic model in axial lumbar MR images using Intervertebral disc Descriptor (IdD). Multimed Tools Appl. 2018;77:27215–27230.

[29] Bevevino AJ, Dickens JF, Potter BK, et al. A model to predict limb salvage in severe combat-related open calcaneus fractures. Clin Orthop Relat Res. 2014;472:3002–3009.

[30] Bien N, Rajpurkar P, Ball RL, et al. Deep-learning-assisted diagnosis for knee magnetic resonance imaging: Development and retrospective validation of MRNet. PLoS Med. 2018;15:e1002699.

[31] Bini SA, Shah RF, Bendich I, et al. Machine Learning Algorithms Can Use Wearable Sensor Data to Accurately Predict Six-Week Patient-Reported Outcome Scores Following Joint Replacement in a Prospective Trial. J Arthroplasty. 2019;34:2242–2247.

[32] Bishop JB, Szpalski M, Ananthraman SK, et al. Classification of low back pain from dynamic motion characteristics using an artificial neural network. Spine (Phila Pa 1976). 1997;22:2991–2998.

[33] Bloomfield RA, Williams HA, Broberg JS, et al. Machine Learning Groups Patients by Early Functional Improvement Likelihood Based on Wearable Sensor Instrumented Preoperative Timed-Up-and-Go Tests. J Arthroplasty. 2019;34:2267–2271.

[34] Boniatis I, Costaridou L, Cavouras D, et al. A morphological descriptors-based pattern recognition system for the characterization of hip osteoarthritis severity from X-ray images. Nucl Instruments Methods Phys Res Sect A Accel Spectrometers, Detect Assoc Equip. 2007;580:1093–1096.

[35] Boniatis I, Costaridou L, Cavouras D, et al. Assessing hip osteoarthritis severity utilizing a probabilistic neural network based classification scheme. Med Eng Phys. 2007;29:227–237.

[36] Burns JE, Yao J, Summers RM. Vertebral body compression fractures and bone density: Automated detection and classification on CT Images. Radiology. 2017;284:788–797.

[37] Cafri G, Li L, Paxton EW, et al. Predicting risk for adverse health events using random forest. J Appl Stat. 2018;45:2279–2294.

[38] Cafri G, Graves SE, Sedrakyan A, et al. Postmarket surveillance of arthroplasty device components using machine learning methods. Pharmacoepidemiol Drug Saf. 2019;28:1440–1447.

[39] Calle-Alonso F, Pérez CJ, Arias-Nicolás JP, et al. Computer-aided diagnosis system: A Bayesian hybrid classification method. Comput Methods Programs Biomed. 2013;112:104–113.

[40] Carballido-Gamio J, Yu A, Wang L, et al. Hip Fracture Discrimination Based on Statistical Multi-parametric Modeling (SMPM). Ann Biomed Eng. 2019;47:2199–2212.

[41] Cerveri P, Belfatto A, Baroni G, et al. Stacked sparse autoencoder networks and statistical shape models for automatic staging of distal femur trochlear dysplasia. Int J Med Robot Comput Assist Surg. 2018;14:e1947.

[42] Chalmers E, Pedrycz W, Lou E. Human experts’ and a fuzzy model’s predictions of outcomes of scoliosis treatment: A comparative analysis. IEEE Trans Biomed Eng. 2015;62:1001–1007.

[43] Chanda S, Gupta S, Pratihar DK. A combined neural network and genetic algorithm based approach for optimally designed femoral implant having improved primary stability. Appl Soft Comput J. 2015;38:296–307.

[44] Chanda S, Gupta S, Pratihar DK. Effects of interfacial conditions on shape optimization of cementless hip stem: an investigation based on a hybrid framework. Struct Multidiscip Optim. 2016;53:1143–1155.

[45] Chang PD, Wong TT, Rasiej MJ. Deep Learning for Detection of Complete Anterior Cruciate Ligament Tear. J Digit Imaging. 2019;32:980–986.

[46] Chang YT, Lin J, Shieh JS, et al. Optimization the initial weights of artificial neural networks via genetic algorithm applied to hip bone fracture prediction. Adv Fuzzy Syst. 2012;

[47] Chatterjee S, Dey S, Majumder S, et al. Computational intelligence based design of implant for varying bone conditions. Int j numer method biomed eng. 2019;35.

[48] Chen P, Gao L, Shi X, et al. Fully automatic knee osteoarthritis severity grading using deep neural networks with a novel ordinal loss. Comput Med Imaging Graph. 2019;75:84–92.

[49] Chen YF, Lin CS, Wang KA, et al. Design of a clinical decision support system for fracture prediction using imbalanced dataset. J Healthc Eng. 2018;2018:9621640.

[50] Cheng CT, Ho TY, Lee TY, et al. Application of a deep learning algorithm for detection and visualization of hip fractures on plain pelvic radiographs. Eur Radiol. 2019;29:5469–5477.

[51] Cho J sung, Cho YS, Moon SB, et al. Scoliosis Screening through a Machine Learning Based Gait Analysis Test. Int J Precis Eng Manuf. 2018;19:1861–1872.

[52] Chung SW, Han SS, Lee JW, et al. Automated detection and classification of the proximal humerus fracture by using deep learning algorithm. Acta Orthop. 2018;89:468–473.

[53] Cilla M, Borgiani E, Martínez J, et al. Machine learning techniques for the optimization of joint replacements: Application to a short-stem hip implant. PLoS One. 2017;12:e0183755.

[54] Couteaux V, Si-Mohamed S, Nempont O, et al. Automatic knee meniscus tear detection and orientation classification with Mask-RCNN. Diagn Interv Imaging. 2019;100:235–242.

[55] Cui S, Zhao L, Wang Y, et al. Using Naive Bayes Classifier to predict osteonecrosis of the femoral head with cannulated screw fixation. Injury. 2018;49:1865–1870.

[56] D’Lima DD, Patil S, Steklov N, et al. ’Lab’-in-a-Knee: In vivo knee forces, kinematics, and contact analysis. Clin Orthop Relat Res. 2011;469:2953–2970.

[57] de Bruijn B, Cranney A, O’Donnell S, et al. Identifying Wrist Fracture Patients with High Accuracy by Automatic Categorization of X-ray Reports. J Am Med Informatics Assoc. 2006;13:696–698.

[58] Dejnabadi H, Jolles BM, Aminian K. A new approach for quantitative analysis of inter-joint coordination during gait. IEEE Trans Biomed Eng. 2008;55:755–764.

[59] Devikanniga D, Joshua Samuel Raj R. Classification of osteoporosis by artificial neural network based on monarch butterfly optimisation algorithm. Healthc Technol Lett. 2018;5:70–75.

[60] Duong L, Cheriet F, Labelle H. Three-dimensional classification of spinal deformities using fuzzy clustering. Spine (Phila Pa 1976). 2006;31:923–930.

[61] Duong L, Cheriet F, Labelle H. Automatic detection of scoliotic curves in posteroanterior radiographs. IEEE Trans Biomed Eng. 2010;57:1143–1151.

[62] Durand WM, Depasse JM, Daniels AH. Predictive modeling for blood transfusion after adult spinal deformity surgery. Spine (Phila Pa 1976). 2018;43:1058–1066.

[63] Elkin PL, Schlegel DR, Anderson M, et al. Artificial Intelligence: Bayesian versus Heuristic Method for Diagnostic Decision Support. Appl Clin Inform. 2018;9:432–439.

[64] Eller-Vainicher C, Chiodini I, Santi I, et al. Recognition of morphometric vertebral fractures by artificial neural networks: Analysis from gismo Lombardia database. PLoS One. 2011;6:e27277.

[65] England JR, Gross JS, White EA, et al. Detection of traumatic pediatric elbow joint effusion using a deep convolutional neural network. Am J Roentgenol. 2018;211:1361–1368.

[66] F.A. H, S. S, I. V de M, et al. Qualitative versus quantitative lumbar spinal stenosis grading by machine learning supported texture analysis-Experience from the LSOS study cohort. Eur J Radiol. 2019;114:45–50.

[67] Fan B, Li HX, Hu Y. An Intelligent Decision System for Intraoperative Somatosensory Evoked Potential Monitoring. IEEE Trans Neural Syst Rehabil Eng. 2016;24:300–307.

[68] Fanfoni CM, Forero FC, Sanches MAA, et al. Evaluation of scoliosis using baropodometer and artificial neural network. Res Biomed Eng. 2017;33:121–129.

[69] Fontana MA, Lyman S, Sarker GK, et al. Can machine learning algorithms predict which patients will achieve minimally clinically important differences from total joint arthroplasty? Clin Orthop Relat Res. 2019;477:1262–1266.

[70] Forestier G, Petitjean F, Riffaud L, et al. Automatic matching of surgeries to predict surgeons’ next actions. Artif Intell Med. 2017;81:3–11.

[71] Forsberg JA, Healey JH, Brennan MF. A probabilistic analysis of completely excised high-grade soft tissue sarcomas of the extremity: An application of a Bayesian belief network. Ann Surg Oncol. 2012;19:2992–3001.

[72] Frighetto-Pereira L, Rangayyan RM, Metzner GA, et al. Shape, texture and statistical features for classification of benign and malignant vertebral compression fractures in magnetic resonance images. Comput Biol Med. 2016;73:147–156.

[73] Gabriel RA, Sharma BS, Doan CN, et al. A Predictive Model for Determining Patients Not Requiring Prolonged Hospital Length of Stay after Elective Primary Total Hip Arthroplasty. Anesth Analg. 2019;129:43–50.

[74] Gan K, Xu D, Lin Y, et al. Artificial intelligence detection of distal radius fractures: a comparison between the convolutional neural network and professional assessments. Acta Orthop. 2019;90:394–400.

[75] García-Cano E, Arámbula Cosío F, Duong L, et al. Dynamic ensemble selection of learner-descriptor classifiers to assess curve types in adolescent idiopathic scoliosis. Med Biol Eng Comput. 2018;56:2221–2231.

[76] Ghaneei M, Ekyalimpa R, Westover L, et al. Customized k-nearest neighbourhood analysis in the management of adolescent idiopathic scoliosis using 3D markerless asymmetry analysis. Comput Methods Biomech Biomed Engin. 2019;22:696–705.

[77] Gowd AK, Agarwalla A, Amin NH, et al. Construct validation of machine learning in the prediction of short-term postoperative complications following total shoulder arthroplasty. J Shoulder Elb Surg. 2019;28:e410–e421.

[78] Goyal A, Ngufor C, Kerezoudis P, et al. Can machine learning algorithms accurately predict discharge to nonhome facility and early unplanned readmissions following spinal fusion? Analysis of a national surgical registry. J Neurosurg Spine. 2019;31:568–578.

[79] Grigsby J, Kooken R, Hershberger J. Simulated neural networks to predict outcomes, costs, and length of stay among orthopedic rehabilitation patients. Arch Phys Med Rehabil. 1994;75:1077–1081.

[80] Hafezi-Nejad N, Guermazi A, Roemer FW, et al. Prediction of medial tibiofemoral compartment joint space loss progression using volumetric cartilage measurements: Data from the FNIH OA biomarkers consortium. Eur Radiol. 2017;27:464–473.

[81] Han SS, Azad TD, Suarez PA, et al. A machine learning approach for predictive models of adverse events following spine surgery. Spine J. 2019;19:1772–1781.

[82] Han Z, Wei B, Leung S, et al. Automated Pathogenesis-Based Diagnosis of Lumbar Neural Foraminal Stenosis via Deep Multiscale Multitask Learning. Neuroinformatics. 2018;16:325–337.

[83] Hao S, Jiang J, Guo Y, et al. Active learning based intervertebral disk classification combining shape and texture similarities. Neurocomputing. 2013;101:252–257.

[84] Hareendranathan AR, Zonoobi D, Mabee M, et al. Semiautomatic classification of acetabular shape from three-dimensional ultrasound for diagnosis of infant hip dysplasia using geometric features. Int J Comput Assist Radiol Surg. 2017;12:439–447.

[85] Harris AHS, Kuo AC, Weng Y, et al. Can Machine Learning Methods Produce Accurate and Easy-to-use Prediction Models of 30-day Complications and Mortality after Knee or Hip Arthroplasty? Clin Orthop Relat Res. 2019;477:452–460.

[86] Hetherington J, Lessoway V, Gunka V, et al. SLIDE: automatic spine level identification system using a deep convolutional neural network. Int J Comput Assist Radiol Surg. 2017;12:1189–1198.

[87] Hoffman H, Lee SI, Garst JH, et al. Use of multivariate linear regression and support vector regression to predict functional outcome after surgery for cervical spondylotic myelopathy. J Clin Neurosci. 2015;22:1444–1449.

[88] Hopkins BS, Weber KA, Kesavabhotla K, et al. Machine Learning for the Prediction of Cervical Spondylotic Myelopathy: A Post Hoc Pilot Study of 28 Participants. World Neurosurg. 2019;127:e436–e442.

[89] Hu B, Kim C, Ning X, et al. Using a deep learning network to recognise low back pain in static standing. Ergonomics. 2018;61:1374–1381.

[90] Huang ZY, Huang C, Xie JW, et al. Analysis of a large data set to identify predictors of blood transfusion in primary total hip and knee arthroplasty. Transfusion. 2018;58:1855–1862.

[91] Huber M, Kurz C, Leidl R. Predicting patient-reported outcomes following hip and knee replacement surgery using supervised machine learning. BMC Med Inform Decis Mak. 2019;19:3.

[92] Hussain D, Han SM. Computer-aided osteoporosis detection from DXA imaging. Comput Methods Programs Biomed. 2019;173:87–107.

[93] Hyer JM, Ejaz A, Tsilimigras DI, et al. Novel Machine Learning Approach to Identify Preoperative Risk Factors Associated with Super-Utilization of Medicare Expenditure Following Surgery. JAMA Surg. 2019;154:1014–1021.

[94] Jamaludin A, Lootus M, Kadir T, et al. ISSLS PRIZE IN BIOENGINEERING SCIENCE 2017: Automation of reading of radiological features from magnetic resonance images (MRIs) of the lumbar spine without human intervention is comparable with an expert radiologist. Eur Spine J. 2017;26:1374–1383.

[95] Jamaludin A, Kadir T, Zisserman A. SpineNet: Automated classification and evidence visualization in spinal MRIs. Med Image Anal. 2017;41:63–73.

[96] Jaremko J, Delorme S, Dansereau J, et al. Use of neural networks to correlate spine and rib deformity in scoliosis. Comput Methods Biomech Biomed Engin. 2000;3:203–213.

[97] Jaremko JL, Poncet P, Ronsky J, et al. Estimation of spinal deformity in scoliosis from torso surface cross sections. Spine (Phila Pa 1976). 2001;26:1583–1591.

[98] Jaremko JL, Poncet P, Ronsky J, et al. Comparison of cobb angles measured manually, calculated from 3-D spinal reconstruction, and estimated from torso asymmetry. Comput Methods Biomech Biomed Engin. 2002;5:277–281.

[99] Jaremko JL, Poncet P, Ronsky J, et al. Genetic algorithm-neural network estimation of Cobb angle from torso asymmetry in scoliosis. J Biomech Eng. 2002;124:496–503.

[100] Jiang P, Missoum S, Chen Z. Fusion of clinical and stochastic finite element data for hip fracture risk prediction. J Biomech. 2015;48:4043–4052.

[101] Jin C, Yang Y, Xue ZJ, et al. Automated analysis method for screening knee osteoarthritis using medical infrared thermography. J Med Biol Eng. 2013;33:471–477.

[102] Jo C, Ko S, Shin WC, et al. Transfusion after total knee arthroplasty can be predicted using the machine learning algorithm. Knee Surgery, Sport Traumatol Arthrosc. 2020;28:1757–1764.

[103] Jones GG, Kotti M, Wiik A V., et al. Gait comparison of unicompartmental and total knee arthroplasties with healthy controls. Bone Jt J. 2016;98-B:16–21.

[104] Kadhim MA. FNDSB: A fuzzy-neuro decision support system for back pain diagnosis. Cogn Syst Res. 2018;52:691–700.

[105] Kalagara S, Eltorai AEM, Durand WM, et al. Machine learning modeling for predicting hospital readmission following lumbar laminectomy. J Neurosurg Spine. 2019;30:344–352.

[106] Karabulut EM, Ibrikci T. Effective Automated Prediction of Vertebral Column Pathologies Based on Logistic Model Tree with SMOTE Preprocessing. J Med Syst. 2014;38:50.

[107] Karamehmetoglu SS, Ugur M, Arslan YZ, et al. A quantitative skin impedance test to diagnose spinal cord injury. Eur Spine J. 2009;18:972–977.

[108] Karhade A V., Ogink PT, Thio QCBS, et al. Machine learning for prediction of sustained opioid prescription after anterior cervical discectomy and fusion. Spine J. 2019;19:976–983.

[109] Karhade A V., Ogink PT, Thio QCBS, et al. Development of machine learning algorithms for prediction of prolonged opioid prescription after surgery for lumbar disc herniation. Spine J. 2019;19:1764–1771.

[110] Karhade A V., Ogink P, Thio Q, et al. Development of machine learning algorithms for prediction of discharge disposition after elective inpatient surgery for lumbar degenerative disc disorders. Neurosurg Focus. 2018;45:E6.

[111] Karhade A V., Schwab JH, Bedair HS. Development of Machine Learning Algorithms for Prediction of Sustained Postoperative Opioid Prescriptions After Total Hip Arthroplasty. J Arthroplasty. 2019;34:2272-2277.e1.

[112] Karhade A V., Shah AA, Bono CM, et al. Development of machine learning algorithms for prediction of mortality in spinal epidural abscess. Spine J. 2019;19:1950–1959.

[113] Karnuta JM, Navarro SM, Haeberle HS, et al. Bundled Care for Hip Fractures: A Machine-Learning Approach to an Untenable Patient-Specific Payment Model. J Orthop Trauma. 2019;33:324–330.

[114] Karnuta JM, Navarro SM, Haeberle HS, et al. Predicting Inpatient Payments Prior to Lower Extremity Arthroplasty Using Deep Learning: Which Model Architecture Is Best? J Arthroplasty. 2019;34:2235-2241.e1.

[115] Kim DH, MacKinnon T. Artificial intelligence in fracture detection: transfer learning from deep convolutional neural networks. Clin Radiol. 2018;73:439–445.

[116] Kim JS, Arvind V, Oermann EK, et al. Predicting Surgical Complications in Patients Undergoing Elective Adult Spinal Deformity Procedures Using Machine Learning. Spine Deform. 2018;6:762–770.

[117] Kim JS, Merrill RK, Arvind V, et al. Examining the Ability of Artificial Neural Networks Machine Learning Models to Accurately Predict Complications Following Posterior Lumbar Spine Fusion. Spine (Phila Pa 1976). 2018;43:853–860.

[118] Kim K, Kim S, Lee YH, et al. Performance of the deep convolutional neural network based magnetic resonance image scoring algorithm for differentiating between tuberculous and pyogenic spondylitis. Sci Rep. 2018;8.

[119] Kitamura G, Chung CY, Moore BE. Ankle Fracture Detection Utilizing a Convolutional Neural Network Ensemble Implemented with a Small Sample, De Novo Training, and Multiview Incorporation. J Digit Imaging. 2019;32:672–677.

[120] Kluge F, Hannink J, Pasluosta C, et al. Pre-operative sensor-based gait parameters predict functional outcome after total knee arthroplasty. Gait Posture. 2018;66:194–200.

[121] Koh J, Chaudhary V, Dhillon G. Disc herniation diagnosis in MRI using a CAD framework and a two-level classifier. Int J Comput Assist Radiol Surg. 2012;7:861–869.

[122] Kotti M, Duffell LD, Faisal AA, et al. Detecting knee osteoarthritis and its discriminating parameters using random forests. Med Eng Phys. 2017;43:19–29.

[123] Krishnaraj A, Barrett S, Bregman-Amitai O, et al. Simulating Dual-Energy X-Ray Absorptiometry in CT Using Deep-Learning Segmentation Cascade. J Am Coll Radiol. 2019;16:1473–1479.

[124] Kruse C, Eiken P, Vestergaard P. Clinical fracture risk evaluated by hierarchical agglomerative clustering. Osteoporos Int. 2017;28:819–832.

[125] Kuo CY, Yu LC, Chen HC, et al. Comparison of models for the prediction of medical costs of spinal fusion in Taiwan diagnosis-related groups by machine learning algorithms. Healthc Inform Res. 2018;24:29–37.

[126] Kwon SB, Ro DH, Song MK, et al. Identifying key gait features associated with the radiological grade of knee osteoarthritis. Osteoarthr Cartil. 2019;27:1755–1760.

[127] Labbe DR, de Guise JA, Mezghani N, et al. Objective grading of the pivot shift phenomenon using a support vector machine approach. J Biomech. 2011;44:1–5.

[128] Lai DTH, Levinger PT, Begg RK, et al. Automatic recognition of gait patterns exhibiting patellofemoral pain syndrome using a support vector machine approach. IEEE Trans Inf Technol Biomed. 2009;13:810–817.

[129] Lee HK, Jin R, Feng Y, et al. An Analytical Framework for TJR Readmission Prediction and Cost-Effective Intervention. IEEE J Biomed Heal Informatics. 2019;23:1760–1772.

[130] Levinger P, Lai DTH, Begg RK, et al. The application of support vector machines for detecting recovery from knee replacement surgery using spatio-temporal gait parameters. Gait Posture. 2009;29:91–96.

[131] Lin CC, Ou YK, Chen SH, et al. Comparison of artificial neural network and logistic regression models for predicting mortality in elderly patients with hip fracture. Injury. 2010;41:869–873.

[132] Lin H. Identification of spinal deformity classification with total curvature analysis and artificial neural network. IEEE Trans Biomed Eng. 2008;55:376–382.

[133] Lindsey R, Daluiski A, Chopra S, et al. Deep neural network improves fracture detection by clinicians. Proc Natl Acad Sci U S A. 2018;115:11591–11596.

[134] Liu Q, Cui X, Chou YC, et al. Ensemble artificial neural networks applied to predict the key risk factors of hip bone fracture for elders. Biomed Signal Process Control. 2015;21:146–156.

[135] Lu HY, Huang CY, Su CT, et al. Predicting rotator cuff tears using data mining and bayesian likelihood ratios. PLoS One. 2014;9:e94917.

[136] Malek S, Gunalan R, Kedija SY, et al. Random forest and Self Organizing Maps application for analysis of pediatric fracture healing time of the lower limb. Neurocomputing. 2018;272:55–62.

[137] Malik OA, Senanayake SMNA, Zaheer D. An Intelligent Recovery Progress Evaluation System for ACL Reconstructed Subjects Using Integrated 3-D Kinematics and EMG Features. IEEE J Biomed Heal Informatics. 2015;19:453–463.

[138] Mandal I. Developing new machine learning ensembles for quality spine diagnosis. Knowledge-Based Syst. 2015;73:298–310.

[139] Mann NH, Brown MD, Hertz DB, et al. Initial-impression diagnosis using low-back pain patient pain drawings. Spine (Phila Pa 1976). 1993;18:41–53.

[140] Mathew B, Norris D, Mackintosh I, et al. Artificial intelligence in the prediction of operative findings in low back surgery. Br J Neurosurg. 1989;3:161–170.

[141] Mehta SD, Sebro R. Computer-Aided Detection of Incidental Lumbar Spine Fractures from Routine Dual-Energy X-Ray Absorptiometry (DEXA) Studies Using a Support Vector Machine (SVM) Classifier. J Digit Imaging. 2020;33:204–210.

[142] Melton LJ, Atkinson EJ, St. Sauver JL, et al. Predictors of excess mortality after fracture: A population-based cohort study. J Bone Miner Res. 2014;29:1681–1690.

[143] Merali ZG, Witiw CD, Badhiwala JH, et al. Using a machine learning approach to predict outcome after surgery for degenerative cervical myelopathy. PLoS One. 2019;14:e0215133.

[144] Merrill RK, Ferrandino RM, Hoffman R, et al. Machine Learning Accurately Predicts Short-Term Outcomes Following Open Reduction and Internal Fixation of Ankle Fractures. J Foot Ankle Surg. 2019;58:410–416.

[145] Mezghani N, Chav R, Humbert L, et al. A computer-based classifier of three-dimensional spinal scoliosis severity. Int J Comput Assist Radiol Surg. 2008;3:55–60.

[146] Mezghani N, Phan P, Mitiche A, et al. A Kohonen neural network description of scoliosis fused regions and their corresponding Lenke classification. Int J Comput Assist Radiol Surg. 2012;7:257–264.

[147] Milimonfared R, Oskouei RH, Taylor M, et al. An intelligent system for image-based rating of corrosion severity at stem taper of retrieved hip replacement implants. Med Eng Phys. 2018;61:13–24.

[148] Minciullo L, Parkes MJ, Felson DT, et al. Comparing image analysis approaches versus expert readers: The relation of knee radiograph features to knee pain. Ann Rheum Dis. 2018;77:1606–1609.

[149] Moustakidis SP, Theocharis JB, Giakas G. A fuzzy decision tree-based SVM classifier for assessing osteoarthritis severity using ground reaction force measurements. Med Eng Phys. 2010;32:1145–1160.

[150] Muehlematter UJ, Mannil M, Becker AS, et al. Vertebral body insufficiency fractures: detection of vertebrae at risk on standard CT images using texture analysis and machine learning. Eur Radiol. 2019;29:2207–2217.

[151] Nam KH, Seo I, Kim DH, et al. Machine learning model to predict osteoporotic spine with hounsfield units on lumbar computed tomography. J Korean Neurosurg Soc. 2019;62:442–449.

[152] Nault ML, Labelle H, Aubin CÉ, et al. Fuzzy-logic-assisted surgical planning in adolescent idiopathic scoliosis. J Spinal Disord Tech. 2009;22:263–269.

[153] Navarro SM, Wang EY, Haeberle HS, et al. Machine Learning and Primary Total Knee Arthroplasty: Patient Forecasting for a Patient-Specific Payment Model. J Arthroplasty. 2018;33:3617–3623.

[154] Nelson AE, Fang F, Arbeeva L, et al. A machine learning approach to knee osteoarthritis phenotyping: data from the FNIH Biomarkers Consortium. Osteoarthr Cartil. 2019;27:994–1001.

[155] Nishiyama KK, Macdonald HM, Hanley DA, et al. Women with previous fragility fractures can be classified based on bone microarchitecture and finite element analysis measured with HR-pQCT. Osteoporos Int. 2013;24:1733–1740.

[156] Norman B, Pedoia V, Noworolski A, et al. Applying Densely Connected Convolutional Neural Networks for Staging Osteoarthritis Severity from Plain Radiographs. J Digit Imaging. 2019;32:471–477.

[157] Ogink PT, Karhade A V., Thio QCBS, et al. Predicting discharge placement after elective surgery for lumbar spinal stenosis using machine learning methods. Eur Spine J. 2019;

[158] Ogink PT, Karhade A V., Thio QCBS, et al. Development of a machine learning algorithm predicting discharge placement after surgery for spondylolisthesis. Eur Spine J. 2019;28:1775–1782.

[159] Oh E, Seo SW, Yoon YC, et al. Prediction of pathologic femoral fractures in patients with lung cancer using machine learning algorithms: Comparison of computed tomography-based radiological features with clinical features versus without clinical features. J Orthop Surg. 2017;25.

[160] Oktay AB, Albayrak NB, Akgul YS. Computer aided diagnosis of degenerative intervertebral disc diseases from lumbar MR images. Comput Med Imaging Graph. 2014;38:613–619.

[161] Olczak J, Fahlberg N, Maki A, et al. Artificial intelligence for analyzing orthopedic trauma radiographs: Deep learning algorithms—are they on par with humans for diagnosing fractures? Acta Orthop. 2017;88:581–586.

[162] Orozco Villaseñor DA, Wimmer MA. Wear Scar Similarities between Retrieved and Simulator-Tested Polyethylene TKR Components: An Artificial Neural Network Approach. Biomed Res Int. 2016;2016:2071945.

[163] Ottenbacher KJ, Linn RT, Smith PM, et al. Comparison of logistic regression and neural network analysis applied to predicting living setting after hip fracture. Ann Epidemiol. 2004;14:551–559.

[164] Pauly O, Diotte B, Fallavollita P, et al. Machine learning-based augmented reality for improved surgical scene understanding. Comput Med Imaging Graph. 2015;41:55–60.

[165] Pedoia V, Lee J, Norman B, et al. Diagnosing osteoarthritis from T2 maps using deep learning: an analysis of the entire Osteoarthritis Initiative baseline cohort. Osteoarthr Cartil. 2019;27:1002–1010.

[166] Pedoia V, Norman B, Mehany SN, et al. 3D convolutional neural networks for detection and severity staging of meniscus and PFJ cartilage morphological degenerative changes in osteoarthritis and anterior cruciate ligament subjects. J Magn Reson Imaging. 2019;49:400–410.

[167] Phan P, Mezghani N, Wai EK, et al. Artificial neural networks assessing adolescent idiopathic scoliosis: Comparison with Lenke classification. Spine J. 2013;13:1527–1533.

[168] Prakash M, Joukainen A, Torniainen J, et al. Near-infrared spectroscopy enables quantitative evaluation of human cartilage biomechanical properties during arthroscopy. Osteoarthr Cartil. 2019;27:1235–1243.

[169] Raghavendra U, Bhat NS, Gudigar A, et al. Automated system for the detection of thoracolumbar fractures using a CNN architecture. Futur Gener Comput Syst. 2018;85:184–189.

[170] Ramirez L, Durdle NG, Raso VJ, et al. A support vector machines classifier to assess the severity of idiopathic scoliosis from surface topography. IEEE Trans Inf Technol Biomed. 2006;10:84–91.

[171] Ramkumar PN, Karnuta JM, Navarro SM, et al. Preoperative Prediction of Value Metrics and a Patient-Specific Payment Model for Primary Total Hip Arthroplasty: Development and Validation of a Deep Learning Model. J Arthroplasty. 2019;34:2228-2234.e1.

[172] Ramkumar PN, Karnuta JM, Navarro SM, et al. Deep Learning Preoperatively Predicts Value Metrics for Primary Total Knee Arthroplasty: Development and Validation of an Artificial Neural Network Model. J Arthroplasty. 2019;34:2220-2227.e1.

[173] Ramkumar PN, Navarro SM, Haeberle HS, et al. Development and Validation of a Machine Learning Algorithm After Primary Total Hip Arthroplasty: Applications to Length of Stay and Payment Models. J Arthroplasty. 2019;34:632–637.

[174] Roblot V, Giret Y, Bou Antoun M, et al. Artificial intelligence to diagnose meniscus tears on MRI. Diagn Interv Imaging. 2019;100:243–249.

[175] Sadatsafavi M, Moayyeri A, Soltani A, et al. Artificial neural networks in prediction of bone density among post-menopausal women. J Endocrinol Invest. 2005;28:425–431.

[176] Saif AFM, Shahnaz C, Zhu WP, et al. Abnormality Detection in Musculoskeletal Radiographs Using Capsule Network. IEEE Access. 2019;7:81494–81503.

[177] Sanders NW, Mann NH. Automated scoring of patient pain drawings using artificial neural networks: Efforts toward a low back pain triage application. Comput Biol Med. 2000;30:287–298.

[178] Sapthagirivasan V, Anburajan M. Diagnosis of osteoporosis by extraction of trabecular features from hip radiographs using support vector machine: An investigation panorama with DXA. Comput Biol Med. 2013;43:1910–1919.

[179] Saygılı A, Albayrak S. An efficient and fast computer-aided method for fully automated diagnosis of meniscal tears from magnetic resonance images. Artif Intell Med. 2019;97:118–130.

[180] Scheer JK, Smith JS, Schwab F, et al. Development of a preoperative predictive model for major complications following adult spinal deformity surgery. J Neurosurg Spine. 2017;26:736–743.

[181] Schwartz MH, Ward RE, Macwilliam C, et al. Using Neural Networks to Identify Patients Unlikely to Achieve a Reduction in Bodily Pain after Total Hip Replacement Surgery. Med Care. 1997;35:1020–1030.

[182] Şen Köktaş N, Yalabik N, Yavuzer G, et al. A multi-classifier for grading knee osteoarthritis using gait analysis. Pattern Recognit Lett. 2010;31:898–904.

[183] Sezer A, Basri Sezer H. Convolutional neural network based diagnosis of bone pathologies of proximal humerus. Neurocomputing. 2020;392:124–131.

[184] Shah AA, Karhade A V., Bono CM, et al. Development of a machine learning algorithm for prediction of failure of nonoperative management in spinal epidural abscess. Spine J. 2019;19:1657–1665.

[185] Shamir L, Ling SM, Scott W, et al. Early detection of radiographic knee osteoarthritis using computer-aided analysis. Osteoarthr Cartil. 2009;17:1307–1312.

[186] Shi L, Wang XC, Wang YS. Artificial neural network models for predicting 1-year mortality in elderly patients with intertrochanteric fractures in China. Brazilian J Med Biol Res. 2013;46:993–999.

[187] Siccoli A, de Wispelaere MP, Schröder ML, et al. Machine learning-based preoperative predictive analytics for lumbar spinal stenosis. Neurosurg Focus. 2019;46:E5.

[188] Silver AE, Lungren MP, Johnson ME, et al. Using support vector machines to optimally classify rotator cuff strength data and quantify post-operative strength in rotator cuff tear patients. J Biomech. 2006;39:973–979.

[189] Staartjes VE, de Wispelaere MP, Vandertop WP, et al. Deep learning-based preoperative predictive analytics for patient-reported outcomes following lumbar discectomy: feasibility of center-specific modeling. Spine J. 2019;19:853–861.

[190] Stojadinovic A, Potter BK, Eberhardt J, et al. Development of a prognostic naïve Bayesian classifier for successful treatment of nonunions. J Bone Jt Surg - Ser A. 2011;93:187–194.

[191] Su FC, Wu WL. Design and testing of a genetic algorithm neural network in the assessment of gait patterns. Med Eng Phys. 2000;22:67–74.

[192] Su Y, Kwok TCY, Cummings SR, et al. Can Classification and Regression Tree Analysis Help Identify Clinically Meaningful Risk Groups for Hip Fracture Prediction in Older American Men (The MrOS Cohort Study)? JBMR Plus. 2019;3.

[193] Subramoniam M, Barani S, Rajini V. A non-invasive computer aided diagnosis of osteoarthritis from digital x-ray images. Biomed Res. 2015;26:721–729.

[194] Swenson ER, Bastian ND, Nembhard HB. Data analytics in health promotion: Health market segmentation and classification of total joint replacement surgery patients. Expert Syst Appl. 2016;60:118–129.

[195] Tay B, Hyun JK, Oh S. A machine learning approach for specification of spinal cord injuries using fractional anisotropy values obtained from diffusion tensor images. Comput Math Methods Med. 2014;2014:276589.

[196] Tighe P, Laduzenski S, Edwards D, et al. Use of Machine Learning Theory to Predict the Need for Femoral Nerve Block Following ACL Repair. Pain Med. 2011;12:1566–1575.

[197] Tomita N, Cheung YY, Hassanpour S. Deep neural networks for automatic detection of osteoporotic vertebral fractures on CT scans. Comput Biol Med. 2018;98:8–15.

[198] Unal Y, Polat K, Erdinc Kocer H. Pairwise FCM based feature weighting for improved classification of vertebral column disorders. Comput Biol Med. 2014;46:61–70.

[199] Urakawa T, Tanaka Y, Goto S, et al. Detecting intertrochanteric hip fractures with orthopedist-level accuracy using a deep convolutional neural network. Skeletal Radiol. 2019;48:239–244.

[200] Valentinitsch A, Trebeschi S, Kaesmacher J, et al. Opportunistic osteoporosis screening in multi-detector CT images via local classification of textures. Osteoporos Int. 2019;30:1275–1285.

[201] Van de Meulebroucke C, Beckers J, Corten K. What Can We Expect Following Anterior Total Hip Arthroplasty on a Regular Operating Table? A Validation Study of an Artificial Intelligence Algorithm to Monitor Adverse Events in a High-Volume, Nonacademic Setting. J Arthroplasty. 2019;34:2260–2266.

[202] Veronezi CCD, De Azevedo Simões PWT, Dos Santos RL, et al. Análise computacional para auxílio ao diagnóstics de osteoartrite de coluna lombar baseado em redes neurais artificial. Rev Bras Ortop. 2011;46:195–199.

[203] Wang N, Huang X, Rao Y, et al. A Convenient Non-harm Cervical Spondylosis Intelligent Identity method based on Machine Learning. Sci Rep. 2018;8:17430.

[204] Wang SQ, Li X, Cui JL, et al. Prediction of myelopathic level in cervical spondylotic myelopathy using diffusion tensor imaging. J Magn Reson Imaging. 2015;41:1682–1688.

[205] Wang S, Hu Y, Shen Y, et al. Classification of Diffusion Tensor Metrics for the Diagnosis of a Myelopathic Cord Using Machine Learning. Int J Neural Syst. 2018;28:1750036.

[206] Watari R, Osis S, Ferber R. Use of baseline pelvic acceleration during running for classifying response to muscle strengthening treatment in patellofemoral pain: A preliminary study. Clin Biomech. 2018;57:74–80.

[207] WeiKoh J, Tan TS, EnChuah Z, et al. Genetic algorithm optimized back propagation neural network for knee osteoarthritis classification. Res J Appl Sci Eng Technol. 2014;8:1787–1793.

[208] Werner FW, Wang H, Short WH, et al. Identifying scapholunate ligamentous injury. J Orthop Res. 2009;27:394–399.

[209] Whiteside D, Martini DN, Lepley AS, et al. Predictors of ulnar collateral ligament reconstruction in major league baseball pitchers. Am J Sports Med. 2016;44:2202–2209.

[210] Wu HY, Gong CSA, Lin SP, et al. Predicting postoperative vomiting among orthopedic patients receiving patient-controlled epidural analgesia using SVM and LR. Sci Rep. 2016;6:27041.

[211] Wu W, Zeng W, Ma L, et al. Modeling and classification of gait patterns between anterior cruciate ligament deficient and intact knees based on phase space reconstruction, Euclidean distance and neural networks. Biomed Eng Online. 2018;17:165.

[212] Xue Y, Zhang R, Deng Y, et al. A preliminary examination of the diagnostic value of deep learning in hip osteoarthritis. PLoS One. 2017;12:e0178992.

[213] Yao J, Burns JE, Muñoz H, et al. Cortical shell unwrapping for vertebral body abnormality detection on computed tomography. Comput Med Imaging Graph. 2014;38:628–638.

[214] Yoo TK, Kim DW, Choi SB, et al. Simple Scoring System and Artificial Neural Network for Knee Osteoarthritis Risk Prediction: A Cross-Sectional Study. PLoS One. 2016;11:e0148724.

[215] Yoo TK, Kim SK, Choi SB, et al. Interpretation of movement during stair ascent for predicting severity and prognosis of knee osteoarthritis in elderly women using support vector machine. Proc Annu Int Conf IEEE Eng Med Biol Soc EMBS. 2013;2013:192–196.

[216] Yu X, Ye C, Xiang L. Application of artificial neural network in the diagnostic system of osteoporosis. Neurocomputing. 2016;214:376–381.

[217] Zeng W, Ismail SA, Pappas E. Classification of gait patterns in patients with unilateral anterior cruciate ligament deficiency based on phase space reconstruction, Euclidean distance and neural networks. Soft Comput. 2019;

[218] Zeng W, Ismail SA, Lim YP, et al. Classification of Gait Patterns Using Kinematic and Kinetic Features, Gait Dynamics and Neural Networks in Patients with Unilateral Anterior Cruciate Ligament Deficiency. Neural Process. Lett. School of Physics and Mechanical and Electrical Engineering, Longyan University, Longyan, 364012, China: Springer New York LLC; 2019. p. 887–909.

[219] Zeng W, Ma L, Yuan C, et al. Classification of asymptomatic and osteoarthritic knee gait patterns using gait analysis via deterministic learning. Artif Intell Rev. 2019;52:449–467.

[220] Zhang G, Smith BP, Plate JF, et al. A systematic approach to predicting the risk of unicompartmental knee arthroplasty revision. Osteoarthr Cartil. 2016;24:991–999.

[221] Zhang J, Li H, Lv L, et al. Computer-Aided Cobb Measurement Based on Automatic Detection of Vertebral Slopes Using Deep Neural Network. Int J Biomed Imaging. 2017;2017:9083916.

[222] Zhang M, Gong H, Zhang K, et al. Prediction of lumbar vertebral strength of elderly men based on quantitative computed tomography images using machine learning. Osteoporos Int. 2019;30:2271–2282.

Some articles were published online ahead of print and came up in the initial search. Their 2020 publication date reflects their print publication date.
