# Supplementary material for: Artificial intelligence in orthopaedics: A scoping review
Source: PLoS One. 2021 Nov 23;16(11):e0260471. doi: 10.1371/journal.pone.0260471 (PMC8610245; doi:10.1371/journal.pone.0260471)
Supplement: Attachment — Submitted filename: Response to reviewers.docx [file pone.0260471.s005.docx]

*PLOS ONE*

16^th^ August 2021

Dear Editor and Reviewers,

Thank you for your time reviewing and commenting on our submission. Please see the table below detailing where specific comments have been addressed in the revised manuscript.

| Introduction   - There should be a concrete example of implementation of AI based decision support or image classification/segmentation to exemplary show the scope of new developments in this field. | First paragraph of introduction  Line 42 |
| --- | --- |
| - Additionally, the ethical and data privacy aspect of saving and processing patient data in big quantities should be addressed. | 2^nd^ paragraph of introduction  Line 52 |
| - Since the European General Data Protection Regulation, medical registries are struggling to gather data and therefore, there is actually a decrease of registry data in most of the European countries. Please take that into account and adjust to it. | 2^nd^ paragraph of introduction  Line 52 |
| Methods   - It is unfortunate that the review stops at 2019, since the quantity of new papers covering this area of interest hast evolved since then. This should be discussed later. | Mentioned in discussion. Line 357 |
| - The exclusion criteria seem very specific and the decision for exclusion should be explained concretely. I.e. exclusion rehabilitation and prosthetics can be applied in clinical application. | Line 95 |
| - Was there full agreement on the studies selected between both authors? Please add whether there was full agreement or if there was agreement negotiated | Line 100 |
| - In table 2 you mention search-terms for MEDLINE but did not report literature search in medline for this section. Please adjust. | Line 79 |
| - Please describe whether you have only searched for articles in English or any other language. | Line 78 and 90 |
| Results   - Short and focused on the few results given. It would be interesting to correlate the year of publication and geographical location of the papers, maybe a graph could be added to emphasize this. | Stacked bar chart as updated figure 3 |
| Discussion   - A clear differentiation between classic algorithms and machine learning or artificial intelligence based self-learning systems needs to be implemented, since this is topic to controversial discussion, since we lack clear definitions to the term “artificial intelligence”. | First paragraph of results. Line 120 |
| - Overlapping research fields: In conclusion this presents us with the opportunity to promote interdisciplinary research, an opportunity which thus far is underdeveloped, especially because specialist knowledge from data science is needed to progress in the field of machine learning and AI in the general field of medicine. | Added to discussion. Line 236. |
| - Generally, I would prefer better structuring of the discussion. I would not put sub-headings and the discussion should be related to the results presented. Unfortunately, in my opinion, a clear concept of this paper is lacking. | Acknowledged. Headings removed. Restructured. |
| - You are mentioning that research of AI in orthopaedics is at an early stage. How can you support this? Is there more research in other medical fields? | Line 186. |
| - In my opinion, parts of the discussion should be rather pointed out in the results section (e.g. line 141-149). Please adjust this to the whole manuscript (strictly describing results versus discussion of these findings). | Results & discussion restructured |
| - Overlapping research fields: this sub-section seems a bit out of place. Unfortunately, I cannot identify the central idea behind this. | Acknowledged. Results & discussion restructured |
| - Please discuss the arising data protection problems in regards of registry data. | Discussed in introduction and again in line 319-322. |
| - Please add references to support your statements (e.g. line 159-161) | References added. Now lines 192-195. |
| Conclusion   - Ethical considerations and data privacy, especially with “big data”, as needed for development of AI and ML (machine learning) need to be discussed. Line 207: “AI in clinical practice is embryoic” -> well put, but the research in this paper is not able to support this thesis well enough and it seems like an overdramatization. Other wording should be considered. | Ethical considerations added. Lines 319-322. Conclusion amended. |
| - The conclusion given make general suggestions, that whilst very interessting, cannot be supported by a scoping review, with the few data end points extracted from the 222 papers included. More careful evaluation of results and more focused discussion and conclusion should be considered | Conclusion amended to be more focused. |
| - In my opinion, your conclusion is more a discussion than a straight conclusion. Please adjust this and identify actual discussion of findings, compared to a straight conclusion based on your manuscript. Furthermore, I think you should point out the relevance of your findings more extensively. | Conclusion amended. |

We hope you find our revised manuscript suitable for publication and look forward to hearing from you in due course.

Yours sincerely,

Simon Federer

MSk Lab

Department of Surgery & Cancer

Imperial College London

Email: simon.federer@nhs.net
