# Supplementary material for: Artificial intelligence in orthopaedics: A scoping review
Source: PLoS One. 2021 Nov 23;16(11):e0260471. doi: 10.1371/journal.pone.0260471 (PMC8610245; doi:10.1371/journal.pone.0260471)
Supplement: Attachment — Submitted filename: Response to reviewers.docx [file pone.0260471.s006.docx]

*PLOS ONE*

8^th^ October 2021

Dear Editor and Reviewers,

Thank you for your time reviewing and commenting on our submission. The further comments and suggestions have been noted, and we have amended our submission accordingly.

Lines 118-122 have been updated with the appropriate terminology. Lines 245-248 have been amended to suggest “Machine learning” as a more suitable umbrella term.

We hope you find our revised manuscript suitable for publication and look forward to hearing from you in due course.

Yours sincerely,

Simon Federer

MSk Lab

Department of Surgery & Cancer

Imperial College London

Email: simon.federer@nhs.net
